# Successive Refinement for Lossy Compression of Individual Sequences

**DOI:** 10.3390/e27040370

**Published:** 2025-03-31

**Authors:** Neri Merhav

**Affiliations:** The Viterbi Faculty of Electrical and Computer Engineering, Technion–Israel Institute of Technology, Technion City, Haifa 3200003, Israel; merhav@ee.technion.ac.il; Tel.: +972-4-8294737

**Keywords:** finite-state machine, Lempel-Ziv algorithm, successive refinement, multiple description coding

## Abstract

We consider the problem of successive-refinement coding for lossy compression of individual sequences, namely, compression in two stages, where in the first stage, a coarse description at a relatively low rate is sent from the encoder to the decoder, and in the second stage, an additional coding rate is allocated in order to refine the description and thereby improve the reproduction. Our main result is in establishing outer bounds (converse theorems) for the rate region where we limit the encoders to be finite-state machines in the spirit of Ziv and Lempel’s 1978 model. The matching achievability scheme is conceptually straightforward. We also consider the more general multiple description coding problem on a similar footing and propose achievability schemes that are analogous to the well-known El Gamal–Cover and the Zhang–Berger achievability schemes of memoryless sources and additive distortion measures.

## 1. Introduction

The notion of *successive refinement* of information refers to systems in which the reconstruction of the source occurs in multiple stages. In these systems, a single encoder encodes the source and communicates with either one decoder or multiple decoders in a step-by-step manner. At each stage, the encoder transmits a portion of the source information to the corresponding decoder, which also has access to all previous transmissions. Each decoder uses all available transmissions to reconstruct the source, possibly incorporating additional side information. The quality of the reconstruction at each stage (or by each decoder) is evaluated based on a predefined distortion measure. One of the motivations of this hierarchical structure is to allow scalability that meets the available channel resources of the various users who receive the compressed information or even to adapt to time-varying channel conditions of a single user. Several studies have addressed the successive refinement problem for probabilistic sources, most notably, memoryless sources, see, e.g., [1], where necessary and sufficient conditions for simultaneously achieving the rate-distortion function at all stages [2,3], where the rate-distortion region was fully characterized in general, and [4], where source coding error exponents were derived. In some later works, such as [5,6,7,8], successive refinement coding was also considered with the incorporation of side information. Successive-refinement coding is also an important special case of the so called *multiple description coding*, which in its simplest form, consists of two encoders that send two different individual descriptions of the source to two separate respective decoders (that do not cooperate with each other), and the compressed bit-streams pertaining to those descriptions are also combined and sent to yet another decoder, whose role is to produce a better reconstruction than both of those of the two individual decoders. The problem of fully characterizing the rate-distortion region of the multiple description coding problem is open in its general form, and there are certain inner and outer bounds to this region, see Chapter 13 of [9] for some details and references therein.

In this paper, we focus on successive refinement of information in the context of individual sequences, namely, deterministic source sequences, as opposed to the traditional setting of random sequences governed by a certain probabilistic mechanism. In that sense, this work can be viewed as an additional step in the development of multiuser information theory for individual sequences, following a series of earlier works of this flavor, initiated by Ziv and Lempel in [10,11,12,13], and continued by others in many articles, such as [14,15,16,17,18,19]. In particular, we consider the problem of successive-refinement coding for lossy compression of individual sequences in two stages, where in the first stage, a coarse description at a relatively low rate is sent from the encoder to the decoder, and in the second stage, an additional coding rate is allocated in order to refine the description and thereby improve the reproduction. Our main results are in establishing outer bounds (converse theorems) for the rate region with individual sequences, where we limit the encoders to be finite-state machines similarly as in [11,12,13] and others. The compatible achievability scheme is conceptually straightforward, and so, we believe that the deeper and more interesting part of the contribution of this work is in the converse theorems, namely, in the outer bounds. Our results are formulated and proved for two stages of coding, but their extension to any fixed number of stages is straightforward. These results can also be viewed as an extension of the fixed-distortion results of [14] to successive-refinement coding.

We also consider the more general multiple description coding problem and propose achievability schemes that are analogous to the well-known El Gamal–Cover [20] and the Zhang–Berger [21] achievability schemes of memoryless sources and additive distortion measures. There is a clear parallelism between the rate expressions that we obtain and those of [20,21], including those that are associated with the gaps between the outer bound and the corresponding inner bounds.

The outline of the remaining part of this paper is as follows. In Section 2, we establish notation conventions and formulate the problem setting. In Section 3, we provide some general background on the LZ algorithm as well as on its conditional form. Section 4 is devoted to the above-mentioned successive refinement outer bound, and finally, in Section 5, we address the more general multiple description problem.

## 2. Notation Conventions and Problem Formulation

Throughout the paper, random variables will be denoted by capital letters, specific values they may take will be denoted by the corresponding lower case letters and their alphabets will be denoted by calligraphic letters. Random vectors, their realizations, and their alphabets will be denoted, respectively, by capital letters, the corresponding lower case letters, and the corresponding calligraphic letters, all superscripted by their dimensions. More specifically, for a given positive integer, *n*, the source vector $\left(x_{1},x_{2},\ldots ,x_{n}\right)$, with components, $x_{i}$, i=1,2,…,n, from a finite-alphabet, X, will be denoted by $x^{n}$. The set of all such *n*-vectors will be denoted by $X^{n}$, which is the *n*–th order Cartesian power of the single-letter source alphabet, X. Likewise, reproduction vectors of length *n*, such as $\left({\hat{x}}_{1},\ldots ,{\hat{x}}_{n}\right)$ and $\left({\tilde{x}}_{1},\ldots ,{\tilde{x}}_{n}\right)$, with components, ${\hat{x}}_{i}$ and ${\tilde{x}}_{i}$, i=1,…,n, from finite-alphabets, $\hat{X}$ and $\tilde{X}$, will be denoted by ${\hat{x}}^{n}\in {\hat{X}}^{n}$ and ${\tilde{x}}^{n}\in {\tilde{X}}^{n}$, respectively. An infinite source sequence $\left(x_{1},x_{2},\ldots \right)$ will be denoted by *x*. The cardinalities of X, $\hat{X}$, and $\tilde{X}$ will be denoted by α, β, and γ, respectively. The value α=∞ is allowed, to incorporate countably infinite and continuous source alphabets. By contrast, β and γ will always be finite.

For two positive integers *i* and *j*, where i≤j, the notation $x_{i}^{j}$ will be used to denote the substring $\left(x_{i},x_{i+1},\ldots ,x_{j}\right)$. For i=1, the subscript ‘1’ will be omitted, and so, the shorthand notation of $\left(x_{1},x_{2},\ldots ,x_{n}\right)$ will be $x^{n}$, as mentioned before. Similar conventions will apply to other sequences. Probability distributions will be denoted by the letter *P* with possible subscripts, depending on the context. The indicator function for an event A will be denoted by I{A}, that is, I{A}=1 is A occurs, and I{A}=0, if not. The logarithmic function, logx, will be understood to be defined to the base 2. Logarithms to the base *e* will be denoted by ln. Let $d_{1}:X^{n}\times {\hat{X}}^{n}\to R^{+}$ and $d_{2}:X^{n}\times {\tilde{X}}^{n}\to R^{+}$ be two arbitrary distortion functions between source vectors, $x^{n}$, and corresponding reproduction vectors, ${\hat{x}}^{n}$ and ${\tilde{x}}^{n}$, respectively.

The successive refinement encoder model is as follows. It is composed of a cascade of two encoders: a reproduction encoder (namely, a vector quantizer) followed by a lossless encoder. The input to the reproduction encoder is the source sequence $x^{n}$ and the output is a pair of reproduction vectors, $\left({\hat{x}}^{n},{\tilde{x}}^{n}\right)\in {\hat{X}}^{n}\times {\tilde{X}}^{n}$ that obeys the distortion constraints, $d_{1}\left(x^{n},{\hat{x}}^{n}\right)\leq nD_{1}$ and $d_{2}\left(x^{n},{\tilde{x}}^{n}\right)\leq nD_{2}$, where $D_{1}\geq 0$ and $D_{2}\geq 0$ are prescribed normalized distortion levels. There are no particular restrictions imposed on the distortion functions. We denote

(1) $$B\left(x^{n}\right)=\left\{\left({\hat{x}}^{n},{\tilde{x}}^{n}\right):\;d_{1}\left(x^{n},{\hat{x}}^{n}\right)\leq nD_{1},\;d_{2}\left(x^{n},{\tilde{x}}^{n}\right)\leq nD_{2}\right\}.$$

The mapping $X^{n}\to {\hat{X}}^{n}\times {\tilde{X}}^{n}$, employed by the reproduction encoder, is arbitrary and not limited. The pair $\left({\hat{x}}^{n},{\tilde{x}}^{n}\right)$ serves as an input to the lossless encoder.

As for the lossless encoder, we follow the same modeling approach as in [11], but with a few adjustments to make it suitable to successive refinement. In particular, the lossless encoder is defined by a set

$$E=(\hat{X},\tilde{X},U,V,S,Z,f_{1},f_{2},g_{1},g_{2}),$$

where $\hat{X}$ and $\tilde{X}$ are as before; U and V are two sets of variable-length binary strings, which both include the empty string λ of length zero; S and Z are two sets of states, each one containing *q* states; $f_{1}:S\times \hat{X}\to U$ and $f_{2}:Z\times \hat{X}\times \tilde{X}\to V$ are the encoder output functions, and finally, $g_{1}:S\times \hat{X}\to S$ and $g_{2}:Z\times \hat{X}\times \tilde{X}\to Z$ are two next-state functions. When the lossless encoder is fed by a sequence of pairs $\left({\hat{x}}_{1},{\tilde{x}}_{1}\right),\left({\hat{x}}_{2},{\tilde{x}}_{2}\right),\ldots$, it outputs a corresponding sequence of pairs of binary strings, $\left(u_{1},v_{1}\right),\left(u_{2},v_{2}\right),\ldots$ according to the following recursive mechanism. For t=1,2,…:

(2) $$\begin{array}{ccc} u_{t} & = & f_{1}\left(s_{t},{\hat{x}}_{t}\right) \end{array}$$

(3) $$\begin{array}{ccc} s_{t+1} & = & g_{1}\left(s_{t},{\hat{x}}_{t}\right) \end{array}$$

(4) $$\begin{array}{ccc} v_{t} & = & f_{2}\left(z_{t},{\hat{x}}_{t},{\tilde{x}}_{t}\right) \end{array}$$

(5) $$\begin{array}{ccc} z_{t+1} & = & g_{2}\left(z_{t},{\hat{x}}_{t},{\tilde{x}}_{t}\right), \end{array}$$

where the initial states, $s_{1}$ and $z_{1}$, are assumed arbitrary fixed members of S and Z, respectively. Similarly as in [11], we adopt the extended notation, of $f_{1}\left(s_{1},{\hat{x}}^{n}\right)$ for $u^{n}$, $g_{1}\left(s_{1},{\hat{x}}^{n}\right)$ for $s_{n+1}$, and similar notations associated with $f_{2}$ and $g_{2}$.

An encoder *E* is said to be information lossless if for every positive integer *k*, the vector $\left(s_{1},f_{1}\left(s_{1},{\hat{x}}^{k}\right),g_{1}\left(s_{1},{\hat{x}}^{k}\right)\right)$ uniquely determines ${\hat{x}}^{k}$ and likewise,

$$\left(s_{1},z_{1},f_{1}\left(s_{1},{\hat{x}}^{k}\right),f_{2}\left(z_{1},{\hat{x}}^{k},{\tilde{x}}^{k}\right),g_{1}\left(s_{1},{\tilde{x}}^{k}\right),g_{2}\left(z_{1},{\hat{x}}^{k},{\tilde{x}}^{k}\right)\right)$$

uniquely determines $\left({\hat{x}}^{k},{\tilde{x}}^{k}\right)$. Let E(q) be the set of all information lossless encoders, {E}, with |S|≤q and |Z|≤q.

Given a lossless encoder *E* and a pair of inputs $\left({\hat{x}}^{n},{\tilde{x}}^{n}\right)$, we define

(6) $${\left[\rho_{E}\left({\hat{x}}^{n}\right)\right]}_{1}=\frac{L(u^{n})}{n}=\frac{L[f_{1}\left(s_{1},{\hat{x}}^{n}\right)]}{n}$$

and

(7) $${\left[\rho_{E}\left({\hat{x}}^{n},{\tilde{x}}^{n}\right)\right]}_{2}=\frac{L\left(u^{n}\right)+L\left(v^{n}\right)}{n}=\frac{L\left[f_{1}\left(s_{1},{\hat{x}}^{n}\right)\right]+L\left[f_{2}\left(z_{1},{\hat{x}}^{n},{\tilde{x}}^{n}\right)\right]}{n},$$

where $L\left(u^{n}\right)=\sum_{i=1}^{n}l\left(u_{i}\right)$, $l(u_{i})$ being the length (in bits) of the binary string $u_{i}$, and similarly for $L(v^{n})$. Recall that for the empty string, λ, we define l(λ)=0. The achievable rate region for $x^{n}$ that is associated with *E* is defined as

(8) $$R_{E}\left(x^{n}\right)=\underset{\left({\hat{x}}^{n},{\tilde{x}}^{n}\right)\in B\left(x^{n}\right)}{⋃}\left\{\left(R_{1},R_{2}\right):\;R_{1}\geq {\left[\rho_{E}\left({\hat{x}}^{n}\right)\right]}_{1},R_{1}+R_{2}\geq {\left[\rho_{E}\left({\hat{x}}^{n},{\tilde{x}}^{n}\right)\right]}_{2}\right\},$$

and the *q*-state achievable rate region for $x^{n}$ is defined as

(9) $$R_{q}\left(x^{n}\right)=\underset{E\in E(q)}{⋃}R_{E}\left(x^{n}\right).$$

The rationale behind the union operations in these definitions is that they are two-dimensional set-theoretic analogues of the minimization operations over the appropriate encoders {E} and reproduction vectors, $\left\{{\hat{x}}^{n}\right\}$, that appear for a single coding rate, like in [11,14], as can be noted from the simple relationship:

(10) $$\begin{array}{cc} & \left\{R:\;R\geq \min_{E\in \{\mathrm{all}\;q-\mathrm{state}\;\mathrm{encoders}\}}\min_{\left\{\hat{x}:\;d\left(x^{n},{\hat{x}}^{n}\right)\leq nD\right\}}\rho_{E}\left({\hat{x}}^{n}\right)\right\}\\ = & \underset{E\in \{\mathrm{all}\;q-\mathrm{state}\;\mathrm{encoders}\}}{⋃}\underset{\left\{{\hat{x}}^{n}:\;d\left(x^{n},{\hat{x}}^{n}\right)\leq nD\right\}}{⋃}\left\{R:\;R\geq \rho_{E}\left({\hat{x}}^{n}\right)\right\} \end{array}$$

for a given generic distortion function *d* and distortion level *D*.

For later use, we also define the joint empirical distribution of *ℓ*-blocks of $\left({\hat{x}}_{i\ell +1}^{i\ell +\ell },{\tilde{x}}_{i\ell +1}^{i\ell +\ell }\right)$, ℓ=0,1,…,n/ℓ−1, provided that *ℓ* divides *n*. Specifically, consider the empirical distribution, $\hat{P}=\left\{\hat{P}\left({\hat{x}}^{\ell },{\tilde{x}}^{\ell }\right),\;{\hat{x}}^{\ell }\in {\hat{X}}^{\ell },\;{\tilde{x}}^{\ell }\in {\tilde{X}}^{\ell }\right\}$, of pairs of *ℓ*-vectors, defined as

(11) $$\hat{P}\left({\hat{x}}^{\ell },{\tilde{x}}^{\ell }\right)=\frac{\ell }{n}\sum_{i=0}^{n/\ell -1}I\left\{{\hat{x}}_{i\ell +1}^{i\ell +\ell }={\hat{x}}^{\ell },\;{\tilde{x}}_{i\ell +1}^{i\ell +\ell }={\tilde{x}}^{\ell }\right\},\;\;\;\;{\hat{x}}^{\ell }\in {\hat{X}}^{\ell },\;{\tilde{x}}^{\ell }\in {\tilde{X}}^{\ell }$$

Let $H({\hat{X}}^{\ell },{\tilde{X}}^{\ell })$ denote the joint empirical entropy of an auxiliary pair of random *ℓ*-vectors, $\left({\hat{X}}^{\ell },{\tilde{X}}^{\ell }\right)$, induced by $\hat{P}$, that is,

(12) $$H\left({\hat{X}}^{\ell },{\tilde{X}}^{\ell }\right)=-\sum_{\left({\hat{x}}^{\ell },{\tilde{x}}^{\ell }\right)\in {\hat{X}}^{\ell }\times {\tilde{X}}^{\ell }}\hat{P}\left({\hat{x}}^{\ell },{\tilde{x}}^{\ell }\right)\log\hat{P}\left({\hat{x}}^{\ell },{\tilde{x}}^{\ell }\right).$$

Accordingly, $H({\hat{X}}^{\ell })$ and $H({\tilde{X}}^{\ell }|{\hat{X}}^{\ell })$ will denote the corresponding marginal empirical entropy of ${\hat{X}}^{\ell }$ and the conditional empirical entropy of ${\tilde{X}}^{\ell }$ given ${\hat{X}}^{\ell }$.

Our objective is to provide inner and outer bounds to the achievable rate region and show that they asymptotically coincide in the limit of large *n* followed by a limit of large *q*, in analogy to the asymptotic regime of [11].

## 3. Background

Before the exposition of the main results and their proofs, we revisit key terms and details related to the 1978 version of the LZ algorithm, also known as the LZ78 algorithm [11], which is the central building block in this work. The incremental parsing procedure of the LZ78 algorithm is a sequential parsing process applied to a finite-alphabet input vector, ${\hat{x}}^{n}$. According to this procedure, each new phrase is the shortest string not encountered before as a parsed phrase, except for the potential incompleteness of the last phrase. For instance, the incremental parsing of the vector ${\hat{x}}^{15}=abbabaabbaaabaa$ results in a,b,ba,baa,bb,aa,ab,aa. Let $c({\hat{x}}^{n})$ denote the number of phrases in ${\hat{x}}^{n}$ resulting from the incremental parsing procedure (in the above example, $c({\hat{x}}^{15})=8$). Furthermore, let $LZ({\hat{x}}^{n})$ denote the length of the LZ78 binary compressed code for ${\hat{x}}^{n}$. According to [11], [Theorem 2] the following inequality holds:

(13) $$\begin{array}{ccc} LZ({\hat{x}}^{n}) & \leq & \left[c\left({\hat{x}}^{n}\right)+1\right]\log\left\{2\beta \left[c\left({\hat{x}}^{n}\right)+1\right]\right\}\\ & = & c\left({\hat{x}}^{n}\right)\log\left[c\left({\hat{x}}^{n}\right)+1\right]+c\left({\hat{x}}^{n}\right)\log\left(2\beta \right)+\log\left\{2\beta \left[c\left({\hat{x}}^{n}\right)+1\right]\right\}\\ & = & c\left({\hat{x}}^{n}\right)\log c\left({\hat{x}}^{n}\right)+c\left({\hat{x}}^{n}\right)\log\left[1+\frac{1}{c({\hat{x}}^{n})}\right]+c\left({\hat{x}}^{n}\right)\log\left(2\beta \right)+\log\left\{2\beta \left[c\left({\hat{x}}^{k}\right)+1\right]\right\}\\ & \leq & c\left({\hat{x}}^{n}\right)\log c\left({\hat{x}}^{n}\right)+\log e+\frac{n(\log\beta )\log(2\beta )}{(1-\varepsilon_{n})\log n}+\log\left[2\beta \left(n+1\right)\right]\\ & \overset{▵}{=} & c\left({\hat{x}}^{n}\right)\log c\left({\hat{x}}^{n}\right)+n\cdot \epsilon \left(n\right), \end{array}$$

where we remind that β is the cardinality of $\hat{X}$, and where both $\varepsilon_{n}$ and ϵ(n) tends to zero as n→∞. In other words, the LZ code-length for ${\hat{x}}^{n}$ is upper bounded by an expression whose main term is $c\left({\hat{x}}^{n}\right)\log c\left({\hat{x}}^{n}\right)$. On the other hand, $c\left({\hat{x}}^{n}\right)\log c\left({\hat{x}}^{n}\right)$ is also known to be the main term of a lower bound (see Theorem 1 of [11]) to the shortest code-length attainable by any information lossless finite-state encoder with no more than *q* states, provided that $\log(q^{2})$ is very small compared to $\log c({\hat{x}}^{n})$. In view of these facts, we henceforth refer to $c\left({\hat{x}}^{n}\right)\log c\left({\hat{x}}^{n}\right)$ as the unnormalized *LZ complexity* of ${\hat{x}}^{n}$, whereas the normalized LZ complexity is defined as

(14) $$\rho_{LZ}\left({\hat{x}}^{n}\right)\overset{▵}{=}\frac{c\left({\hat{x}}^{n}\right)\log c\left({\hat{x}}^{n}\right)}{n}.$$

A useful inequality that relates the empirical entropy of non-overlapping *ℓ*-blocks of ${\hat{x}}^{n}$ (where *ℓ* divides *n*) and $\rho_{LZ}\left({\hat{x}}^{n}\right)$ (see, for example, Equation (26) of [22]), is the following:

(15) $$\begin{array}{ccc} \frac{H({\hat{X}}^{\ell })}{\ell } & \geq & \rho_{LZ}\left(x^{n}\right)-\frac{\log[4\beta^{2\ell }]\log\beta }{(1-\varepsilon_{n})\log n}-\frac{\beta^{2\ell }\log\left[4\beta^{2\ell }\right]}{n}-\frac{1}{\ell }\\ & \overset{▵}{=} & \rho_{LZ}\left(x^{n}\right)-\delta_{n}\left(\ell \right), \end{array}$$

It is obtained from the fact that the Shannon code for *ℓ*-blocks can be implemented using a finite-state encoder with no more than $\beta^{\ell }$ states. Specifically, for a block code of length *ℓ* to be implemented by a finite-state machine, one defines the state at each time instant *i* to be the contents of the input, starting at the beginning of the current block (at time ℓ·⌊i/ℓ⌋+1) and ending at time i−1. The number of states for an input alphabet of size β is then $\sum_{i=0}^{\ell -1}\beta^{i}=\left(\beta^{\ell }-1\right)/\left(\beta -1\right)<\beta^{\ell }$. Therefore, the code-length of this Shannon code must comply with the lower bound of Theorem 1 in [11]. Note that $\lim_{n\to \infty }\delta_{n}\left(\ell \right)=1/\ell$ and so, $\lim_{\ell \to \infty }\lim_{n\to \infty }\delta_{n}\left(\ell \right)=0$. Clearly, it is possible to let ℓ=ℓ(n) increase with *n* slowly enough such that $\delta_{n}\left(\ell \left(n\right)\right)\to 0$ as n→∞, in particular, ℓ(n) should be o(logn) for that purpose.

In [22], the notion of the LZ complexity was extended to incorporate finite-state lossless compression in the presence of side information, namely, the conditional version of the LZ complexity. Given ${\hat{x}}^{n}$ and ${\tilde{x}}^{n}$, let us apply the incremental parsing procedure of the LZ algorithm to the sequence of pairs $\left(\left({\hat{x}}_{1},{\tilde{x}}_{1}\right),\left({\hat{x}}_{2},{\tilde{x}}_{2}\right),\ldots ,\left({\hat{x}}_{n},{\tilde{x}}_{n}\right)\right)$. As mentioned before, according to this procedure, all phrases are distinct with a possible exception of the last phrase, which might be incomplete. Let $c({\hat{x}}^{n},{\tilde{x}}^{n})$ denote the number of distinct phrases. As an example (taken from [22]), let n=6 and consider the sequence pair $\left({\hat{x}}^{6},{\tilde{x}}^{6}\right)$ along with its joint incremental parsing as follows:

(16) $$\begin{array}{ccc} {\hat{x}}^{6} & = & 0\;|\;1\;|\;0\;1\;|\;0\;1|\\ {\tilde{x}}^{6} & = & 0\;|\;1\;|\;0\;0\;|\;0\;1| \end{array}$$

then $c({\hat{x}}^{6},{\tilde{x}}^{6})=4$. Let $c^{\prime }\left({\hat{x}}^{n}\right)$ denote the resulting number of distinct phrases of ${\hat{x}}^{k}$ (which may differ from $c({\hat{x}}^{n})$ in individual parsing of ${\hat{x}}^{n}$ alone), and let $\hat{x}\left(l\right)$ denote the *l*-th distinct $\hat{x}$–phrase, $l=1,2,\ldots ,c^{\prime }\left({\hat{x}}^{n}\right)$. In the above example, $c({\hat{x}}^{6})=3$. Denote by $c_{l}\left({\tilde{x}}^{n}|{\hat{x}}^{n}\right)$ the number of occurrences of $\hat{x}\left(l\right)$ in the parsing of ${\hat{x}}^{n}$, or equivalently, the number of distinct $\tilde{x}$-phrases that jointly appear with $\hat{x}\left(l\right)$. Clearly, $\sum_{l=1}^{c^{\prime }\left({\hat{x}}^{n}\right)}c_{l}\left({\tilde{x}}^{n}|{\hat{x}}^{n}\right)=c\left({\hat{x}}^{n},{\tilde{x}}^{n}\right)$. In the above example, $\hat{x}\left(1\right)=0$, $\hat{x}\left(2\right)=1$, $\hat{x}\left(3\right)=01$, $c_{1}\left({\tilde{x}}^{6}|{\hat{x}}^{6}\right)=c_{2}\left({\tilde{x}}^{6}|{\hat{x}}^{6}\right)=1$, and $c_{3}\left({\tilde{x}}^{6}|{\hat{x}}^{6}\right)=2$. Now, the conditional LZ complexity of ${\tilde{x}}^{n}$ given ${\hat{x}}^{n}$ is defined as

(17) $$\rho_{LZ}\left({\tilde{x}}^{n}|{\hat{x}}^{n}\right)\overset{▵}{=}\frac{1}{n}\sum_{l=1}^{c^{\prime }\left({\hat{x}}^{n}\right)}c_{l}\left({\tilde{x}}^{n}|{\hat{x}}^{n}\right)\log c_{l}\left({\tilde{x}}^{n}|{\hat{x}}^{n}\right).$$

In [22] it was shown that $\rho_{LZ}\left({\tilde{x}}^{n}|{\hat{x}}^{n}\right)$ is the main term of the compression ratio achieved by the conditional version of the LZ algorithm for compressing ${\tilde{x}}^{n}$ in the presence of the side information ${\hat{x}}^{n}$, available to both encoder and decoder—see the compression scheme described in [22] (see also [15]), i.e., the length function, $LZ({\tilde{x}}^{n}|{\hat{x}}^{n})$, of the coding scheme proposed therein is upper bounded (in parallel to (13)) by

(18) $$LZ\left({\tilde{x}}^{n}|{\hat{x}}^{n}\right)\leq n\rho_{LZ}\left({\tilde{x}}^{n}|{\hat{x}}^{n}\right)+n\hat{\epsilon }\left(n\right),$$

where $\hat{\epsilon }\left(n\right)=O\left(\frac{\log(\log n)}{\log n}\right)$ (see Equations (10) and (11) in [15]). On the other hand, analogously to [11] [Theorem 1], it was shown in [16], that $\rho_{LZ}\left({\tilde{x}}^{n}|{\hat{x}}^{n}\right)$ is also the main term of a lower bound to the compression ratio that can be achieved by any finite-state encoder with side information at both ends, provided that the number of states is not too large, similarly as described above for the unconditional version.

The inequality (15) also extends to the conditional case as follows (see [16]):

(19) $$\frac{H({\tilde{X}}^{\ell }|{\hat{X}}^{\ell })}{\ell }\geq \rho_{LZ}\left({\tilde{x}}^{n}|{\hat{x}}^{n}\right)-\delta_{n}^{\prime }\left(\ell \right),$$

where $\delta_{n}^{\prime }\left(\ell \right)$ is the same as $\delta_{n}\left(\ell \right)$ except that $\beta^{\ell }$ therein is replaced by ${\left(\beta \gamma \right)}^{\ell }$ to accommodate the number of states associated with the conditional version of the aforementioned Shannon code applied to *ℓ*-blocks. By the same token, we also have

(20) $$\frac{H({\hat{X}}^{\ell },{\tilde{X}}^{\ell })}{\ell }\geq \rho_{LZ}\left({\hat{x}}^{n},{\tilde{x}}^{n}\right)-\delta_{n}^{\prime }\left(\ell \right).$$

## 4. The Outer Bound for Successive Refinement

Our main result for finite-state encoders is the following.

**Theorem** **1.** *For every $x^{n}\in X^{n}$*,

(21) $$\begin{array}{ccc} R_{q}\left(x^{n}\right) & \subseteq & R_{o}\left(x^{n}\right)\\ & \overset{▵}{=} & \underset{\left({\hat{x}}^{n},{\tilde{x}}^{n}\right)\in B\left(x^{n}\right)}{⋃}R_{LZ}\left({\hat{x}}^{n},{\tilde{x}}^{n}\right), \end{array}$$

*where*

(22) $$\begin{array}{ccc} R_{LZ}\left({\hat{x}}^{n},{\tilde{x}}^{n}\right) & \overset{▵}{=} & \{(R_{1},R_{2}):\\ R_{1} & \geq & \rho_{LZ}\left({\hat{x}}^{n}\right)-\Delta_{1}\left(q,n\right),\\ R_{1}+R_{2} & \geq & \rho_{LZ}\left({\hat{x}}^{n}\right)+\rho_{LZ}\left({\tilde{x}}^{n}|{\hat{x}}^{n}\right)-\Delta_{2}\left(q,n\right)\}, \end{array}$$

*where $\Delta_{1}\left(q,n\right)$ and $\Delta_{2}\left(q,n\right)$ are defined as*

(23) $$\Delta_{1}\left(q,n\right)=\frac{\log(4q^{2})\log\beta }{(1-\epsilon_{n})\log n}+\frac{q^{2}\log\left(4q^{2}\right)}{n}$$

*with $\epsilon_{n}\to 0$ as n→∞*, *and*

(24) $$\begin{array}{ccc} \Delta_{2}\left(n,q\right) & = & \min_{\left\{\ell :\;\ell \;divides\;n\right\}}\left\{\delta_{n}\left(\ell \right)+\delta_{n}^{\prime }\left(\ell \right)+\frac{1}{\ell }\log\left[q^{4}\left(1+\log\left[1+\frac{\beta^{\ell }\gamma^{\ell }}{q^{4}}\right]\right)\right]\right\}. \end{array}$$

**Discussion** **1.** *Several comments are now in order*.

*1.*  *Since both $\Delta_{1}\left(q,n\right)$ and $\Delta_{2}\left(q,n\right)$ tend to zero as n→∞ for fixed q, the asymptotic achievability of $R_{o}\left(x^{n}\right)$ is conceptually straightforward: Given an internal point, $\left(R_{1},R_{2}\right)\in R_{o}\left(x^{n}\right)$, there must be at least one pair $\left({\hat{x}}^{n},{\tilde{x}}^{n}\right)\in B\left(x^{n}\right)$ such that $\left(R_{1},R_{2}\right)\in R_{LZ}\left({\hat{x}}^{n},{\tilde{x}}^{n}\right)$. Upon finding such a pair, proceed as follows: At the first stage, apply LZ78 compression to ${\hat{x}}^{n}$ at a coding rate of $R_{1}=\frac{LZ({\hat{x}}^{n})}{n}$ which is only slightly above $\rho_{LZ}\left({\hat{x}}^{n}\right)$ for large n, as discussed in Section 3. At the second stage, apply conditional LZ compression of ${\tilde{x}}^{n}$ given ${\hat{x}}^{n}$ as side information at both ends, at an incremental coding rate of $R_{2}=\frac{LZ({\tilde{x}}^{n}|{\hat{x}}^{n})}{n}$ which is close to $\rho_{LZ}\left({\tilde{x}}^{n}|{\hat{x}}^{n}\right)$, as also explained in Section 3, and then the total rate, $R_{1}+R_{2}$, is about $\rho_{LZ}\left({\hat{x}}^{n}\right)+\rho_{LZ}\left({\tilde{x}}^{n}|{\hat{x}}^{n}\right)$. Similarly as in [11], there is still a certain gap between the achievability and the converse theorem because the achievability requires encoders whose number of states is not small compared to n, whereas the converse is significant when q is very small relative to n. As in [11], this gap can be closed in the asymptotic limit of large q by partitioning the sequence into non-overlapping blocks and starting over the LZ compression mechanism in each block separately. We will address this point in detail later on.*
*2.*  *Considering that the achievability is conceptually straightforward, as explained in item no. 1 above, the interesting and deeper result is the converse theorem. Since the second stage encoder receives both ${\hat{x}}^{n}$ and ${\tilde{x}}^{n}$ as inputs, it is immediate to lower bound the total coding rate, at the second stage, in terms of the joint compressibility of $\left({\hat{x}}^{n},{\tilde{x}}^{n}\right)$, namely by $\rho_{LZ}\left({\hat{x}}^{n},{\tilde{x}}^{n}\right)$, but recall that the first-stage encoder must have already allocated a rate at least as large as $\rho_{LZ}\left({\hat{x}}^{n}\right)$, then in order to meet a lower bound of $\rho_{LZ}\left({\hat{x}}^{n},{\tilde{x}}^{n}\right)$ on the total coding rate, the incremental rate, $R_{2}$, of the second stage must not exceed $\rho_{LZ}\left({\hat{x}}^{n},{\tilde{x}}^{n}\right)-\rho_{LZ}\left({\hat{x}}^{n}\right)$, and there is no apparent way to achieve such a coding rate, as far as the author can see. Nonetheless, since we can also lower bound the total rate of both stages by $\rho_{LZ}\left({\hat{x}}^{n}\right)+\rho_{LZ}\left({\tilde{x}}^{n}|{\hat{x}}^{n}\right)$, then the achievability becomes obvious, as said. This point is not trivial because there is no chain rule that applies to the LZ complexities of arbitrary finite sequences. The proof that $\rho_{LZ}\left({\hat{x}}^{n}\right)+\rho_{LZ}\left({\tilde{x}}^{n}|{\hat{x}}^{n}\right)$ also serves as a lower bound (essentially), which requires a certain manipulation by using a generalized Kraft inequality and passing via empirical entropies, as can be seen in the proof.*
*3.*  *The choice of ${\hat{x}}^{n}$ exhibits a trade-off between the coding rate of the first stage and the incremental rate at the second stage because ${\hat{x}}^{n}$ is both compressed at the first stage and serves as side information at the second stage, so there might be a certain tension between selecting ${\hat{x}}^{n}$ for having small $\rho_{LZ}\left({\hat{x}}^{n}\right)$ and selecting it for small $\rho_{LZ}\left({\tilde{x}}^{n}|{\hat{x}}^{n}\right)$. Of course, an analogous tension exists also in successive refinement for memoryless sources [3]. The reproduction encoder must select $\left({\hat{x}}^{n},{\tilde{x}}^{n}\right)\in B\left(x^{n}\right)$ that best compromises these criteria.*
*4.*  *The results extend straightforwardly to any finite number of stages, where at each stage one applies conditional LZ compression of the current reproduction given all previous reproductions.*

**Proof of Theorem** **1.** We begin with the first stage. By definition, if $\left(R_{1},R_{2}\right)\in R_{q}\left(x^{n}\right)$, then there must exist an encoder E∈E(q) and $\left({\hat{x}}^{n},{\tilde{x}}^{n}\right)\in B\left(x^{n}\right)$ such that $R_{1}\geq {\left[\rho_{E}\left({\hat{x}}^{n}\right)\right]}_{1}$ and $R_{1}+R_{2}\geq {\left[\rho_{E}\left({\hat{x}}^{n},{\tilde{x}}^{n}\right)\right]}_{2}$. Now, according to Theorem 1 of [11]:

(25) $$\begin{array}{ccc} {\left[\rho_{E}\left({\hat{x}}^{n}\right)\right]}_{1} & \geq & \frac{c\left({\hat{x}}^{n}\right)+q^{2}}{n}\cdot \log\left[\frac{c\left({\hat{x}}^{n}\right)+q^{2}}{4q^{2}}\right]+\frac{2q^{2}}{n}\\ & > & \frac{c\left({\hat{x}}^{n}\right)+q^{2}}{n}\cdot \log\left[c\left({\hat{x}}^{n}\right)+q^{2}\right]-\frac{c\left({\hat{x}}^{n}\right)+q^{2}}{n}\log\left(4q^{2}\right)\\ & > & \frac{c\left({\hat{x}}^{n}\right)\log c\left({\hat{x}}^{n}\right)}{n}-\frac{c\left({\hat{x}}^{n}\right)\log\left(4q^{2}\right)}{n}-\frac{q^{2}\log\left(4q^{2}\right)}{n}\\ & \geq & \frac{c\left({\hat{x}}^{n}\right)\log c\left({\hat{x}}^{n}\right)}{n}-\frac{\log(4q^{2})\log\beta }{(1-\epsilon_{n})\log n}-\frac{q^{2}\log\left(4q^{2}\right)}{n}\\ & = & \rho_{LZ}\left({\hat{x}}^{n}\right)-\Delta_{1}\left(q,n\right), \end{array}$$

where $\lim_{n\to \infty }\epsilon_{n}=0$ and the last inequality is an application of Equation (6) in [11]. Since $R_{1}\geq {\left[\rho_{E}\left({\hat{x}}^{n}\right)\right]}_{1}$, it follows that

(26) $$R_{1}\geq \rho_{LZ}\left({\hat{x}}^{n}\right)-\Delta_{1}\left(q,n\right).$$

Moving on to the combined encoder of both stages, consider the following. According to Lemma 2 of [11] and due to the postulated information losslessness, the combined encoder, which has $q^{2}$ states, must obey the following generalized Kraft inequality:

(27) $$\sum_{({\hat{x}}^{\ell },{\tilde{x}}^{\ell })\in {\hat{X}}^{\ell }\times {\tilde{X}}^{\ell }}\exp_{2}\left\{-[\min_{s\in S}L\left[f_{1}\left(s,{\hat{x}}^{\ell }\right)\right]+\min_{z\in Z}L\left[f_{2}\left(z,{\hat{x}}^{\ell },{\tilde{x}}^{\ell }\right)\right]\right\}\leq q^{4}\left(1+\log\left[1+\frac{\beta^{\ell }\gamma^{\ell }}{q^{4}}\right]\right).$$

This implies that the description length at the output of this encoder is lower bounded as follows.

(28) $$\begin{array}{ccc} n(R_{1}+R_{2}) & \geq & n{\left[\rho_{E}\left({\hat{x}}^{n},{\tilde{x}}^{n}\right)\right]}_{2}\\ & = & L\left(u^{n}\right)+L\left(v^{n}\right)\\ & = & \sum_{t=1}^{n}\left\{L\left[f_{1}\left(s_{t},{\hat{x}}_{t}\right)\right]+L\left[f_{2}\left(z_{t}.{\hat{x}}_{t},{\tilde{x}}_{t}\right)\right]\right\}\\ & = & \sum_{m=0}^{n/\ell -1}\sum_{j=1}^{\ell }\left\{L\left[f_{1}\left(s_{m\ell +j},{\hat{x}}_{m\ell +j}\right)\right]+L\left[f_{2}\left(z_{m\ell +j},{\hat{x}}_{m\ell +j}\right),{\tilde{x}}_{m\ell +j}\right)\right]\}\\ & = & \sum_{m=0}^{n/\ell -1}\left\{L\left[f_{1}\left(s_{m\ell +1},{\hat{x}}_{m\ell +1}^{m\ell +\ell }\right)\right]+L\left[f_{2}\left(z_{m\ell +1},{\hat{x}}_{m\ell +1}^{m\ell +\ell },{\tilde{x}}_{m\ell +1}^{m\ell +\ell }\right)\right]\right\}\\ & \geq & \sum_{m=0}^{n/\ell -1}\left\{\min_{s\in S}L\left[f_{1}\left(s,{\hat{x}}_{m\ell +1}^{m\ell +\ell }\right)\right]+\min_{z\in Z}L\left[f_{2}\left(z,{\hat{x}}_{m\ell +1}^{m\ell +\ell },{\tilde{x}}_{m\ell +1}^{m\ell +\ell }\right)\right]\right\}\\ & = & \frac{n}{\ell }\sum_{\left({\hat{x}}^{\ell },{\tilde{x}}^{\ell }\right)\in {\hat{X}}^{\ell }\times {\tilde{X}}^{\ell }}\hat{P}\left({\hat{x}}^{\ell },{\tilde{x}}^{\ell }\right)\cdot \left\{\min_{s\in S}L\left[f_{1}\left(s,{\hat{x}}^{\ell }\right)\right]+\min_{z\in Z}L\left[f_{2}\left(z,{\hat{x}}^{\ell },{\tilde{x}}^{\ell }\right)\right]\right\} \end{array}$$

and so,

(29) $$R_{1}+R_{2}\geq \frac{1}{\ell }\sum_{({\hat{x}}^{\ell },{\tilde{x}}^{\ell })\in {\hat{X}}^{\ell }\times {\tilde{X}}^{\ell }}\hat{P}\left({\hat{x}}^{\ell },{\tilde{x}}^{\ell }\right)\cdot \left\{\min_{s\in S}L\left[f_{1}\left(s,{\hat{x}}^{\ell }\right)\right]+\min_{z\in Z}L\left[f_{2}\left(z,{\hat{x}}^{\ell },{\tilde{x}}^{\ell }\right)\right]\right\}.$$

Now, by the generalized Kraft inequality above,

$$\begin{array}{cc} & q^{4}\left(1+\log\left[1+\frac{\beta^{\ell }\gamma^{\ell }}{q^{4}}\right]\right)\\ \geq & \sum_{\left({\hat{x}}^{\ell },{\tilde{x}}^{\ell }\right)\in {\hat{X}}^{\ell }\times {\tilde{X}}^{\ell }}\exp_{2}\left\{-\left(\min_{s\in S}L\left[f_{1}\left(s,{\hat{x}}^{\ell }\right)\right]+\min_{z\in Z}L\left[f_{2}\left(z,{\hat{x}}^{\ell },{\tilde{x}}^{\ell }\right)\right]\right)\right\}\\ = & \sum_{\left({\hat{x}}^{\ell },{\tilde{x}}^{\ell }\right)\in {\hat{X}}^{\ell }\times {\tilde{X}}^{\ell }}\hat{P}\left({\hat{x}}^{\ell },{\tilde{x}}^{\ell }\right)\cdot \exp_{2}\left\{-\left(\min_{s\in S}L[f_{1}\left(s,{\hat{x}}^{\ell }\right)+\min_{z\in Z}L\left[f_{2}\left(z,{\hat{x}}^{\ell },{\tilde{x}}^{\ell }\right)\right]\right)-\log\hat{P}\left({\hat{x}}^{\ell },{\tilde{x}}^{\ell }\right)\right\}\\ \geq & \exp_{2}\left\{-\sum_{\left({\hat{x}}^{\ell },{\tilde{x}}^{\ell }\right)\in {\hat{X}}^{\ell }\times {\tilde{X}}^{\ell }}\hat{P}\left({\hat{x}}^{\ell },{\tilde{x}}^{\ell }\right)\cdot \left(\min_{s\in S}L[f_{1}\left(s,{\hat{x}}^{\ell }\right)+[\min_{z\in Z}L\left[f_{2}\left(z,{\hat{x}}^{\ell },{\tilde{x}}^{\ell }\right)\right]\right)+H\left({\hat{X}}^{\ell },{\tilde{X}}^{\ell }\right)\right\}, \end{array}$$

where the last inequality follows from the convexity of the exponential function and Jensen’s inequality. This yields

(30) $$\begin{array}{cc} & \log\left\{q^{4}\left(1+\log\left[1+\frac{\beta^{\ell }\gamma^{\ell }}{q^{4}}\right]\right)\right\}\\ \geq & H\left({\hat{X}}^{\ell },{\tilde{X}}^{\ell }\right)-\sum_{\left({\hat{x}}^{\ell },{\tilde{x}}^{\ell }\right)\in {\hat{X}}^{\ell }\times {\tilde{X}}^{\ell }}\hat{P}\left({\hat{x}}^{\ell },{\hat{x}}^{\ell }\right)\cdot \left\{\min_{s\in S}L\left[f_{1}\left(s,{\hat{x}}^{\ell }\right)\right]+\min_{z\in Z}L\left[f_{2}\left(z,{\hat{x}}^{\ell },{\tilde{x}}^{\ell }\right)\right]\right\}, \end{array}$$

implying that

(31) $$\begin{array}{ccc} R_{1}+R_{2} & \geq & \frac{L\left(u^{n}\right)+L\left(v^{n}\right)}{n}\\ & \geq & \frac{1}{\ell }\sum_{\left({\hat{x}}^{\ell },{\tilde{x}}^{\ell }\right)\in {\hat{X}}^{\ell }\times {\tilde{X}}^{\ell }}\hat{P}\left({\hat{x}}^{\ell },{\tilde{x}}^{\ell }\right)\cdot \left\{\min_{s\in S}L\left[f_{1}\left(s,{\hat{x}}^{\ell }\right)\right]+\min_{z\in Z}L\left[f_{2}\left(z,{\hat{x}}^{\ell },{\tilde{x}}^{\ell }\right)\right]\right\}\\ & \geq & \frac{H({\hat{X}}^{\ell },{\tilde{X}}^{\ell })}{\ell }-\frac{1}{\ell }\log\left\{q^{4}\left(1+\log\left[1+\frac{\beta^{\ell }\gamma^{\ell }}{q^{4}}\right]\right)\right\}\\ & = & \frac{H({\hat{X}}^{\ell })}{\ell }+\frac{H({\tilde{X}}^{\ell }|{\hat{X}}^{\ell })}{\ell }-\frac{1}{\ell }\log\left\{q^{4}\left(1+\log\left[1+\frac{\beta^{\ell }\gamma^{\ell }}{q^{4}}\right]\right)\right\} \end{array}$$

Now, according to Equation (15),

(32) $$\frac{H({\hat{X}}^{\ell })}{\ell }\geq \rho_{LZ}\left({\hat{x}}^{n}\right)-\delta_{n}\left(\ell \right).$$

Similarly, according to Equation (19),

(33) $$\frac{H({\tilde{X}}^{\ell }|{\hat{X}}^{\ell })}{\ell }\geq \rho_{LZ}\left({\tilde{x}}^{n}|{\hat{x}}^{n}\right)-\delta_{n}^{\prime }\left(\ell \right),$$

and so,

(34) $$\begin{array}{ccc} R_{1}+R_{2} & \geq & \rho_{LZ}\left({\hat{x}}^{n}\right)+\rho_{LZ}\left({\tilde{x}}^{n}|{\hat{x}}^{n}\right)-\delta_{n}\left(\ell \right)-\delta_{n}^{\prime }\left(\ell \right)-\frac{1}{\ell }\log\left[q^{4}\left(1+\log\left[1+\frac{\beta^{\ell }\gamma^{\ell }}{q^{4}}\right]\right)\right]. \end{array}$$

Maximizing this lower bound w.r.t. *ℓ* yields

(35) $$R_{1}+R_{2}\geq \rho_{LZ}\left({\hat{x}}^{n}\right)+\rho_{LZ}\left({\tilde{x}}^{n}|{\hat{x}}^{n}\right)-\Delta_{2}\left(n,q\right),$$

where

(36) $$\begin{array}{ccc} \Delta_{2}\left(n,q\right) & = & \min_{\left\{\ell :\;\ell \;\mathrm{divides}\;n\right\}}\left\{\delta_{n}\left(\ell \right)+\delta_{n}^{\prime }\left(\ell \right)+\frac{1}{\ell }\log\left[q^{4}\left(1+\log\left[1+\frac{\beta^{\ell }\gamma^{\ell }}{q^{4}}\right]\right)\right]\right\}. \end{array}$$

This completes the proof of Theorem 1. □

Referring to the last part of comment no. 1 in the discussion that follows Theorem 1, we now address the gap in terms of the number of states. For an infinite source sequence $x=(x_{1},x_{2},\ldots )$, we define the *q*-state achievable rate region for *x* as

(37) $$R_{q}\left(x\right)=\underset{m\geq 1}{⋃}\underset{n\geq m}{⋂}R_{q}\left(x^{n}\right),$$

and finally, the finite-state achievable rate region for *x* is defined as

(38) $$R_{\infty }\left(x\right)=\underset{q\geq 1}{⋃}R_{q}\left(x\right).$$

These definitions are two-dimensional counterparts of Equations (2)–(4) in [11], where the finite-state (lossless) compressibility of *x* is defined in several steps. In particular, the union over intersections in the definition of $R_{q}\left(x\right)$ is the set-theoretic analogue of the limit superior operation, and the union operation in the definition of $R_{\infty }\left(x\right)$ is parallel to a limit of q→∞.

Let *k* be a positive integer that divides *n*, and consider the partition of ${\hat{x}}^{n}$ and ${\tilde{x}}^{n}$ into n/k blocks of length *k*, i.e., ${\hat{x}}_{kt+1}^{kt+k}=\left({\hat{x}}_{kt+1},{\hat{x}}_{kt+2},\ldots ,{\hat{x}}_{kt+k}\right)$ and ${\tilde{x}}_{kt+1}^{kt+k}=\left({\tilde{x}}_{kt+1},{\tilde{x}}_{kt+2},\ldots ,{\tilde{x}}_{kt+k}\right)$, t=0,1,…,n/k−1. Next, define:

(39) $$\begin{array}{ccc} R_{-}^{k}\left({\hat{x}}^{n},{\tilde{x}}^{n}\right) & = & \{\left(R_{1},R_{2}\right):\;R_{1}\geq \frac{k}{n}\sum_{t=0}^{n/k-1}\rho_{LZ}\left({\hat{x}}_{kt+1}^{kt+k}\right)-\Delta_{1}\left(q,k\right),\\ & & R_{1}+R_{2}\geq \frac{k}{n}\sum_{t=0}^{n/k-1}\left[\rho_{LZ}\left({\hat{x}}_{kt+1}^{kt+k}\right)+\rho_{LZ}\left({\tilde{x}}_{kt+1}^{kt+k}|{\hat{x}}_{kt+1}^{kt+k}\right)\right]-\Delta_{2}\left(q,k\right)\}. \end{array}$$

Then, similarly as in Theorem 1,

(40) $$R_{q}\left(x^{n}\right)\subseteq R_{o}^{k}\left(x^{n}\right)\overset{▵}{=}\underset{\left({\hat{x}}^{n},{\tilde{x}}^{n}\right)\in B\left(x^{n}\right)}{⋃}R_{-}^{k}\left({\hat{x}}^{n},{\tilde{x}}^{n}\right),$$

and so, for every positive integer *N*:

(41) $$\underset{n\geq N}{⋂}R_{q}\left(x^{n}\right)\subseteq \underset{n\geq N}{⋂}R_{o}^{k}\left(x^{n}\right),$$

implying that

(42) $$R_{q}\left(x\right)=\underset{N\geq 1}{⋃}\underset{n\geq N}{⋂}R_{q}\left(x^{n}\right)\subseteq \underset{N\geq 1}{⋃}\underset{n\geq N}{⋂}R_{o}^{k}\left(x^{n}\right)\overset{▵}{=}R_{o}^{k}\left(x\right).$$

Since this holds for every positive integer *k*, then

(43) $$R_{q}\left(x\right)\subseteq \underset{K\geq 1}{⋃}\underset{k\geq K}{⋂}R_{o}^{k}\left(x\right)\overset{▵}{=}R_{o}\left(x\right),$$

and so,

(44) $$R_{\infty }\left(x\right)\subseteq R_{o}\left(x\right),$$

which establishes an asymptotic version of the converse theorem.

As for the direct part, considering the fact that a block code of length *k*, operating on *k*-tuples of the two reconstruction vectors can be implemented by a finite-state machine with no more than ${\left(\beta \gamma \right)}^{k}$ states, we have

(45) $$\begin{array}{ccc} R_{{\left(\beta \gamma \right)}^{k}}\left(x^{n}\right) & ⊇ & R_{i}^{k}\left(x^{n}\right)\\ & \overset{▵}{=} & \underset{\left({\hat{x}}^{n},{\tilde{x}}^{n}\right)\in B\left(x^{n}\right)}{⋃}R_{+}^{k}\left({\hat{x}}^{n},{\tilde{x}}^{n}\right), \end{array}$$

where

(46) $$\begin{array}{ccc} R_{+}^{k}\left({\hat{x}}^{n},{\tilde{x}}^{n}\right) & = & \{\left(R_{1},R_{2}\right):\;R_{1}\geq \frac{k}{n}\sum_{t=0}^{n/k-1}\rho_{LZ}\left({\hat{x}}_{kt+1}^{kt+k}\right)+O\left(\frac{1}{\log k}\right),\\ & & R_{1}+R_{2}\geq \frac{k}{n}\sum_{t=0}^{n/k-1}[\rho_{LZ}\left({\hat{x}}_{kt+1}^{kt+k}\right)+\\ & & \rho_{LZ}\left({\tilde{x}}_{kt+1}^{kt+k}|{\hat{x}}_{kt+1}^{kt+k}\right))]+O\left(\frac{\log(\log k)}{\log k}\right)\}. \end{array}$$

(47) $$\begin{array}{ccc} R_{{\left(\beta \gamma \right)}^{k}}\left(x\right) & = & \underset{N\geq 1}{⋃}\underset{n\geq N}{⋂}R_{{\left(\beta \gamma \right)}^{k}}\left(x^{n}\right)\\ & ⊇ & \underset{N\geq 1}{⋃}\underset{n\geq N}{⋂}R_{i}^{k}\left(x^{n}\right)\\ & = & R_{i}^{k}\left(x\right)\\ & ⊇ & \underset{K\geq k}{⋂}R_{i}^{K}\left(x\right) \end{array}$$

and so,

(48) $$R_{\infty }\left(x\right)=\underset{k\geq 1}{⋃}R_{{\left(\beta \gamma \right)}^{k}}\left(x\right)⊇\underset{k\geq 1}{⋃}\underset{K\geq k}{⋂}R_{i}^{K}\left(x\right)=R_{i}\left(x\right).$$

We have just proved the following theorem:

**Theorem** **2.** *For every infinite individual sequence $x=(x_{1},x_{2},\ldots )$*,

(49) $$R_{o}\left(x\right)⊇R_{\infty }\left(x\right)⊇R_{i}\left(x\right).$$

These inner and outer bounds are tight in the sense that the definitions of $R_{o}\left(x\right)$ and $R_{i}\left(x\right)$ are based on the same building blocks and the only difference is in terms that tend to zero as k→∞.

## 5. Multiple Description Coding

Consider next the configuration that is associated with the multiple description problem (see, e.g., Chapter 13 of [9]), where the source is an individual sequence and the encoders are modeled as finite-state machines. In particular, there are two *q*-state encoders and three decoders, which are defined as follows. Encoders 1 and 2 are fed by $x^{n}$ and produce two reconstructions, ${\hat{x}}^{n}$ and ${\tilde{x}}^{n}$, with distortions $d_{1}\left(x^{n},{\hat{x}}^{n}\right)\leq nD_{1}$ and $d_{2}\left(x^{n},{\tilde{x}}^{n}\right)\leq nD_{2}$, respectively. Encoder 1 then compresses ${\hat{x}}^{n}$ losslessly and sends a compressed description to Decoder 1. Likewise, Encoder 2 does the same with ${\tilde{x}}^{n}$ and sends a compressed form to Decoder 2. There is no collaboration between Decoders 1 and 2. The third decoder, Decoder 0, receives both compressed descriptions and generates yet another reconstruction, ${\overset{`}{x}}^{n}$, with distortion $d_{0}\left(x^{n},{\overset{`}{x}}^{n}\right)\leq nD_{0}$.

Using the same technique as in the proof of Theorem 1, it is easy to prove the following outer bound to the achievable rate region:

(50) $$R_{o}\left(x^{n}\right)=\underset{\left({\hat{x}}^{n},{\tilde{x}}^{n},{\overset{`}{x}}^{n}\right)\in B\left(x^{n}\right)}{⋃}R_{LZ}\left({\hat{x}}^{n},{\tilde{x}}^{n},{\overset{`}{x}}^{n}\right),$$

where $B(x^{n})$ is redefined as

(51) $$B\left(x^{n}\right)=\left\{\left({\hat{x}}^{n},{\tilde{x}}^{n},{\overset{`}{x}}^{n}\right):\;d_{0}\left(x^{n}{\overset{`}{x}}^{n}\right)\leq nD_{0},\;d_{1}\left(x^{n}{\hat{x}}^{n}\right)\leq nD_{1},\;d_{2}\left(x^{n}{\tilde{x}}^{n}\right)\leq nD_{2}\right\},$$

and

(52) $$\begin{array}{ccc} R_{LZ}\left({\hat{x}}^{n},{\tilde{x}}^{n},{\overset{`}{x}}^{n}\right) & = & \{\left(R_{1},R_{2}\right):\;R_{1}\geq \rho_{LZ}\left({\hat{x}}^{n}\right)-\Delta_{1}\left(q,n\right),\;R_{2}\geq \rho_{LZ}\left({\tilde{x}}^{n}\right)-\Delta_{1}\left(q,n\right),\\ & & R_{1}+R_{2}\geq \rho_{LZ}\left({\overset{`}{x}}^{n}|{\hat{x}}^{n},{\tilde{x}}^{n}\right)+\\ & & \rho_{LZ}\left({\hat{x}}^{n},{\tilde{x}}^{n}\right)-\Delta_{2}\left(q,n\right)\}, \end{array}$$

with $\Delta_{1}\left(q,n\right)$ and $\Delta_{2}\left(q,n\right)$ being defined similarly as before. The sum-rate inequality is obtained by considering that the two encoders together compress losslessly the triple $\left({\hat{x}}^{n},{\tilde{x}}^{n},{\overset{`}{x}}^{n}\right)$ and so, the main term of the lower bound to $R_{1}+R_{2}$ is the joint empirical entropy of of $\left({\hat{x}}^{n},{\tilde{x}}^{n},{\overset{`}{x}}^{n}\right)$, which can be decomposed as the sum of the joint empirical entropy of $\left({\hat{x}}^{n},{\tilde{x}}^{n}\right)$ and the conditional empirical entropy of ${\overset{`}{x}}^{n}$ given $\left({\hat{x}}^{n},{\tilde{x}}^{n}\right)$, which in turn are essentially further lower bounded by $\rho_{LZ}\left({\hat{x}}^{n},{\tilde{x}}^{n}\right)$ and $\rho_{LZ}\left({\overset{`}{x}}^{n}|{\hat{x}}^{n},{\tilde{x}}^{n}\right)$, respectively.

We next present two inner bounds, where the first one is analogous to the El Gamal–Cover inner bound [20] and the second follows the same line of thought as that of the Zhang–Berger inner bound [21].

The former inner bound is given by

(53) $$R_{i}^{EGC}\left(x^{n}\right)=\underset{\left({\hat{x}}^{n},{\tilde{x}}^{n},{\overset{`}{x}}^{n}\right)\in B\left(x^{n}\right)}{⋃}R_{i}\left({\hat{x}}^{n},{\tilde{x}}^{n},{\overset{`}{x}}^{n}\right),$$

where

(54) $$\begin{array}{ccc} R_{i}\left({\hat{x}}^{n},{\tilde{x}}^{n},{\overset{`}{x}}^{n}\right) & = & \{\left(R_{1},R_{2}\right):\;R_{1}\geq \rho_{LZ}\left({\hat{x}}^{n}\right)+\epsilon \left(n\right),\;R_{2}\geq \rho_{LZ}\left({\tilde{x}}^{n}\right)+\epsilon \left(n\right),\\ & & R_{1}+R_{2}\geq \rho_{LZ}\left({\overset{`}{x}}^{n}|{\hat{x}}^{n},{\tilde{x}}^{n}\right)+\rho_{LZ}\left({\hat{x}}^{n},{\tilde{x}}^{n}\right)+\hat{I}\left({\hat{x}}^{n};{\tilde{x}}^{n}\right)+\epsilon \left(n\right)+\hat{\epsilon }\left(n\right)\}, \end{array}$$

where ϵ(n) and $\hat{\epsilon (n)}$ are as in (13) and (18), respectively.

(55) $$\hat{I}\left({\hat{x}}^{n};{\tilde{x}}^{n}\right)=\rho_{LZ}\left({\hat{x}}^{n}\right)+\rho_{LZ}\left({\tilde{x}}^{n}\right)-\rho_{LZ}\left({\hat{x}}^{n},{\tilde{x}}^{n}\right).$$

The quantity $\hat{I}\left({\hat{x}}^{n};{\tilde{x}}^{n}\right)$ plays a role of an empirical mutual information between ${\hat{x}}^{n}$ and ${\tilde{x}}^{n}$, which manifests the gap between the lower bounds to the sum-rate inequalities of the inner bound and the outer bound, analogously to the mutual information term of the El Gamal–Cover achievable region.

The achievability of the above inner bound is as follows. Given an internal point in $R_{i}^{EGC}\left(x^{n}\right)$, there must exist a reconstruction triple $\left({\hat{x}}^{n},{\tilde{x}}^{n},{\overset{`}{x}}^{n}\right)$ that meets the distortion constraints and the corresponding rate inequalities. The encoder applies individual LZ compression for both ${\hat{x}}^{n}$ and ${\tilde{x}}^{n}$ and sends the compressed versions, at rates $\rho_{LZ}\left({\hat{x}}^{n}\right)$ and $\rho_{LZ}\left({\tilde{x}}^{n}\right)$ (up to negligibly small terms for large *n*), to Decoder 1 and Decoder 2, respectively. It then applies conditional LZ compression of ${\overset{`}{x}}^{n}$ given $\left({\hat{x}}^{n},{\tilde{x}}^{n}\right)$ at rate $\rho_{LZ}\left({\overset{`}{x}}^{n}|{\hat{x}}^{n},{\tilde{x}}^{n}\right)$ (up to small terms), and splits this compressed bit stream between Decoders 1 and 2 without violating their rate inequalities. The rate sum is then essentially

(56) $$\begin{array}{cc} & \rho_{LZ}\left({\overset{`}{x}}^{n}|{\hat{x}}^{n},{\tilde{x}}^{n}\right)+\rho_{LZ}\left({\hat{x}}^{n}\right)+\rho_{LZ}\left({\tilde{x}}^{n}\right)\\ = & \rho_{LZ}\left({\overset{`}{x}}^{n}|{\hat{x}}^{n},{\tilde{x}}^{n}\right)+\rho_{LZ}\left({\hat{x}}^{n},{\tilde{x}}^{n}\right)+\hat{I}\left({\hat{x}}^{n};{\tilde{x}}^{n}\right). \end{array}$$

In the above description, we explained that the bit-stream associated with the conditional compression of ${\overset{`}{x}}^{n}$ given $\left({\hat{x}}^{n},{\tilde{x}}^{n}\right)$ is split between Decoders 1 and 2 without violating the rate inequalities. This is always possible because of the following simple fact: Given an internal point in the region $R=\{\left(R_{1},R_{2}\right):\;R_{1}>A,\;R_{2}>B,\;R_{1}+R_{2}\geq A+B+C\}$, there must exist 0≤D≤C such that (A+D,B+C−D)∈R. In particular, let $D=R_{1}-A\geq 0$. Then, $R_{2}=B+C-D\geq B$ and $R_{1}+R_{2}=\left(A+D\right)+\left(B+C-D\right)=A+B+C$. In our case, $A=\rho_{LZ}\left({\hat{x}}^{n}\right)$, $B=\rho_{LZ}\left({\tilde{x}}^{n}\right)$, and $C=\rho_{LZ}\left({\overset{`}{x}}^{n}|{\hat{x}}^{n},{\tilde{x}}^{n}\right)$.

The second achievability scheme, in the spirit of the Zhang–Berger scheme, is as follows. Here, in addition to ${\hat{x}}^{n}$, ${\tilde{x}}^{n}$ and ${\overset{`}{x}}^{n}$, we also generate an auxiliary finite-alphabet sequence, $u^{n}$. The encoder applies LZ compression to $u^{n}$ and conditional LZ compression of ${\hat{x}}^{n}$ given $u^{n}$ and sends both bit-streams to Decoder 1. At the same time, it also applies conditional LZ compression of ${\tilde{x}}^{n}$ given $u^{n}$ and sends the compressed forms of $u^{n}$ and ${\tilde{x}}^{n}$ to Decoder 2. Finally, the encoder applies conditional LZ compression of ${\overset{`}{x}}^{n}$ given $\left({\hat{x}}^{n},{\tilde{x}}^{n},u^{n}\right)$ and splits the compressed bit-stream between Decoders and Decoder 2 in a manner that meets the rate constraints. Thus,

(57) $$\begin{array}{ccc} R_{1} & \approx & \rho_{LZ}\left(u^{n}\right)+\rho_{LZ}\left({\hat{x}}^{n}|u^{n}\right)+\alpha \rho_{LZ}\left({\overset{`}{x}}^{n}|{\hat{x}}^{n},{\tilde{x}}^{n},u^{n}\right) \end{array}$$

(58) $$\begin{array}{ccc} R_{2} & \approx & \rho_{LZ}\left(u^{n}\right)+\rho_{LZ}\left({\tilde{x}}^{n}|u^{n}\right)+\left(1-\alpha \right)\rho_{LZ}\left({\overset{`}{x}}^{n}|{\hat{x}}^{n},{\tilde{x}}^{n},u^{n}\right), \end{array}$$

where α∈[0,1]. Thus,

(59) $$\begin{array}{ccc} R_{1}+R_{2} & \approx & 2\cdot \rho_{LZ}\left(u^{n}\right)+\rho_{LZ}\left({\hat{x}}^{n}|u^{n}\right)+\rho_{LZ}\left({\tilde{x}}^{n}|u^{n}\right)+\rho_{LZ}\left({\overset{`}{x}}^{n}|{\hat{x}}^{n},{\tilde{x}}^{n},u^{n}\right)\\ & = & 2\cdot \rho_{LZ}\left(u^{n}\right)+\rho_{LZ}\left({\hat{x}}^{n},{\tilde{x}}^{n}|u^{n}\right)+\rho_{LZ}\left({\overset{`}{x}}^{n}|{\hat{x}}^{n},{\tilde{x}}^{n},u^{n}\right)+\hat{I}\left({\hat{x}}^{n};{\tilde{x}}^{n}|u^{n}\right), \end{array}$$

where

(60) $$\hat{I}\left({\hat{x}}^{n};{\tilde{x}}^{n}|u^{n}\right)=\rho_{LZ}\left({\hat{x}}^{n}|u^{n}\right)+\rho_{LZ}\left({\tilde{x}}^{n}|u^{n}\right)-\rho_{LZ}\left({\hat{x}}^{n},{\tilde{x}}^{n}|u^{n}\right),$$

is analogous to conditional mutual information. The first term in the rate sum is analogous to 2I(U;X), the sum of the second and the third is analogous to $I(X;\hat{X},\tilde{X},\overset{`}{X}|U)$ and the last term is analogous to $I(\hat{X};\tilde{X}|U)$ (see Theorem 13.4, p. 332 in [9]).

## Data Availability

No new data were created or analyzed in this study. Data sharing is not applicable to this article.
